# Gastrointestinal bacterial infections precede disease activation and treatment intensification in patients with inflammatory bowel disease

**DOI:** 10.1177/17562848251386318

**Published:** 2025-10-22

**Authors:** Merit Kase, Krista Johanna Vitikainen, Clas-Göran af Björkesten, Veli-Jukka Anttila, Leo Meriranta, Perttu Arkkila, Pauliina Molander

**Affiliations:** Department of Internal Medicine and Rehabilitation, Helsinki University Hospital and University of Helsinki, P.O. Box 340 (Haartmaninkatu 4), Helsinki FI-00290, Finland; Department of Surgery, Helsinki University Hospital and University of Helsinki, Helsinki, Finland; Abdominal Center, Gastroenterology, Helsinki University Hospital and University of Helsinki, Helsinki, Finland; Department of Infectious Diseases, Helsinki University Hospital and University of Helsinki, Helsinki, Finland; Department of Oncology, Helsinki University Hospital and University of Helsinki, Helsinki, Finland; Abdominal Center, Gastroenterology, Helsinki University Hospital and University of Helsinki, Helsinki, Finland; Abdominal Center, Gastroenterology, Helsinki University Hospital and University of Helsinki, Helsinki, Finland

**Keywords:** *Campylobacter*, Crohn’s disease, gastrointestinal infection, *Salmonella*, ulcerative colitis, *Yersinia*

## Abstract

**Background:**

Patients with inflammatory bowel disease (IBD) are at heightened risk of infection for several reasons.

**Objectives:**

To evaluate the factors predisposing IBD patients to gastrointestinal (GI) infections, the elements influencing the severity of these infections and the likelihood of hospitalization, and the impact of GI bacterial infections on the progression of IBD.

**Design:**

Retrospective, single-center, case-control study of IBD patients with GI infection and matched controls.

**Methods:**

Patients with ulcerative colitis (UC) or Crohn’s disease who tested positive for *Campylobacter* spp., *Yersinia* spp., *Salmonella* spp., or *enterohemorrhagic Escherichia coli* were enrolled. Clostridioides *difficile* was excluded due to its distinct epidemiology. For each patient with a GI bacterial infection, a control patient with IBD but without the infection was matched by sex and proximity in calendar age. Data were collected from electronic patient records spanning from January 2008 to June 2023. Various risk factors for medical consultations and hospitalizations due to GI bacterial infections were evaluated. Fisher’s exact test and logistic regression were used to assess the associations between GI infections and clinical outcomes.

**Results:**

Of the 5194 IBD patients in the IBD registry, 123 patients had a confirmed GI infection, the majority having UC (*n* = 79, 64%). Patients who experienced GI infection had a higher likelihood of experiencing an IBD flare within 1–6 months post-infection, often requiring intensification of medication, than the control group. However, such factors as age, IBD phenotype, disease activity, comorbidities, IBD pharmacological treatment, and prior travel to countries with lower hygiene standards did not increase the risk of severe bacterial infection or the likelihood of requiring hospitalization.

**Conclusion:**

Our study indicates that IBD patients diagnosed with GI infections may be at elevated risk of experiencing an IBD flare within 1–6 months post-infection, often necessitating the intensification of their pharmacological treatment.

## Plain language summary

Bacterial gut infections can trigger IBD flare-ups

Gastrointestinal (GI) infections such as *Campylobacter*, *Salmonella*, *Yersinia*, and EHEC are common causes of diarrhea. Patients with inflammatory bowel disease (IBD), Crohn’s disease (CD) and ulcerative colitis (UC), are at higher risk of these infections, which may worsen their disease. We studied 123 IBD patients with proven GI infections (2008–2023) and compared them with age- and sex-matched IBD patients without infection.

Main findings:

• 37% of infected patients had a flare within 6 months vs 11% of controls.

• UC patients were hospitalized more often than CD patients.

• Biologics and thiopurines did not raise hospitalization risk.

• Having IBD for less than 2 years doubled infection risk.

**Conclusion:** GI infections can trigger IBD flares. Testing for infection during flares is important, and patients should be monitored for 6 months afterward. Immunosuppressive therapy appears safe.

## Introduction

Inflammatory Bowel Diseases (IBD), including Crohn’s disease (CD) and ulcerative colitis (UC), are chronic inflammatory disorders of the gastrointestinal (GI) tract. Although the exact etiology is not fully understood, IBD is believed to result from a dysregulated mucosal immune response to commensal gut flora in genetically susceptible individuals.^1^ An estimated 2.5–3 million people in Europe are affected by IBD,^2^ with one of the highest prevalence rates worldwide observed in Finland.^3^

The treatment of IBD frequently involves immunosuppressive and immunomodulatory medications,^4–7^ which leave the patients immuno-compromised and more susceptible to infections by common GI pathogens.^8–10^

Enteric pathogens are a leading cause of bacterial diarrhea worldwide, even in high-income regions such as Europe.^11^ Based on estimates from the World Health Organization, unsafe food causes illness in around 600 million people and leads to 420,000 deaths each year, contributing to a loss of 33 million healthy life years (measured in disability-adjusted life years). Although the WHO has not provided an exact figure for bacterial foodborne cases in 2024, current data strongly suggest that bacterial pathogens are responsible for well over half of these illnesses.^12^

In 2022, campylobacteriosis was identified as the most frequently reported zoonosis in Europe, accounting for 61.3% of all confirmed human cases, followed by salmonellosis, yersiniosis, and Shiga toxin-producing *Escherichia coli* infections.^12^ *Campylobacter*, *Yersinia*, *Salmonella*, and *enterohemorrhagic Escherichia coli* (EHEC) are primarily transmitted to humans through the ingestion of contaminated food or water.^11,13,14^ The symptoms vary from watery diarrhea to severe inflammatory diarrhea with abdominal pain, bloody diarrhea and fever, and can be burdened by complications.^11,14^ While these infections typically cause self-limited diarrhea, they may lead to severe or systemic infections in immunocompromised or elderly patients, necessitating antibiotic therapy.^11,14,15^

GI bacterial infections are often associated with IBD flares, making differential diagnosis challenging, as a wide range of inflammatory or infectious diseases can mimic or complicate IBD.^16^ Epidemiological and microbiological studies suggest that enteropathogenic microorganisms play a substantial role in both the initial onset of IBD and in the reactivation of quiescent disease. Consequently, microbiological screening is valuable for patients experiencing IBD flares to guide optimal medical treatment.^10^ While the association between *Clostridioides difficile* infections (CDI) and IBD flares is well established,^17^ the frequency of other enteric infections in relapses of IBD may be underreported, largely due to challenges in detecting enteric pathogens. However, previous studies have indicated a low incidence of non-CDI bacterial infections during IBD,^18^ with GI infections showing no significant negative impact on patient outcomes.^19^

This study aimed to describe the factors that predispose to GI bacterial infections and how these infections present and evolve in patients with IBD. The goal was to identify potential risk factors that may predict the severity and outcomes of GI infections in IBD patients.

## Methods

**Figure 1.**
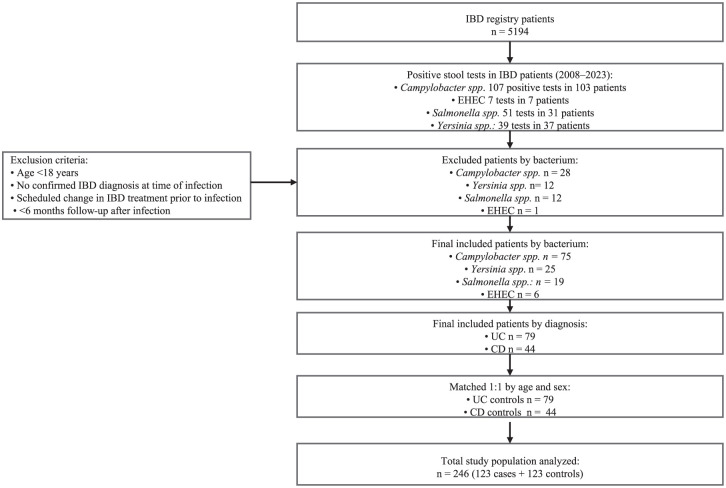
Study flow diagram. Flow of patient inclusion and matching between IBD patients with GI bacterial infections and matched IBD controls. GI, gastrointestinal; IBD, inflammatory bowel disease.

This was a retrospective, observational case-control study in the Hospital District of Helsinki and Uusimaa, which is the largest hospital district in Finland, covering a population of more than 1.7 million. We identified IBD patients with positive stool samples by performing a search combining the positive stool sample registry of the Hospital District and the IBD registry, which is an integrated platform of the hospital patient data system and comprises more than 5000 secondary or tertiary care patients with a confirmed IBD diagnosis, treated mostly with immunosuppressants and biologicals. In Finland, the diagnosis of IBD follows strict international guidelines. It is based on endoscopic evaluation demonstrating macroscopic abnormalities and microscopic changes in biopsy specimens, supported by elevated biomarkers such as fecal calprotectin, with imaging studies used when needed.^20^ All eligible patients were consecutively selected adults (18 years and older) with an established diagnosis of UC or CD and a confirmed GI infection with *Campylobacter* spp., *Salmonella* spp., *Yersinia* spp., or EHEC, based on a positive stool culture and/or nucleic acid test (Amplidiag^®^ Bacterial GE assay (Mobidiag, Espoo, Finland)) analyzed in HUS Diagnostic Center. No IBD patients with *Shigella* infection were identified; thus, infections caused by *Shigella* spp. are not included in this paper. For each patient with a GI bacterial infection, a control patient with IBD but without the infection was selected from the IBD registry, matched for sex and for the closest possible date of birth to ensure comparability in age. The selection of cases and matched controls is shown in the study flow diagram, Figure 1.

### Data collection

Data were collected retrospectively from the electronic patient charts between December 2021 and October 2023 and have been generated as part of standard clinical care and follow-up of IBD patients in Finland. As this was a retrospective study, the sample size was determined by the number of eligible patients meeting the inclusion criteria during the study period. A formal sample size calculation was not performed. For all eligible patients, we collected the following data on possible predisposing factors to an infection: age, sex, pregnancy, IBD type, IBD phenotype,^21^ IBD duration, previous surgical treatment for IBD, IBD pharmacological treatment, other comorbidities (expressed with Charlson Comorbidity Index (CCI)^22^), smoking status, and traveling during 3 months preceding bacterial infection was diagnosed. We also focused on signs and symptoms of GI bacterial infection: fever, abdominal pain, diarrhea, bloody stools, and nausea/vomiting. We investigated the outcomes of bacterial infection, which included antibiotic therapies for bacterial infection, IBD therapy escalation, emergency department visits, and hospitalization. We also examined patient reports up to 6 months after GI infection to monitor IBD activity and assess the need to intensify IBD-related therapy. Those patients whose medication had been planned to be changed before the bacterial infection were excluded from the analyses.

Clinical disease activity before and after the bacterial infection was determined according to the presence or absence of symptoms due to IBD (number of bowel movements, presence or absence of abdominal pain, and presence of blood with defecation). The disease activity was based on the interpretation of patient chart data by two experienced gastroenterologists (original appraiser, P.M.). The reporting of this study conforms to the Strengthening the Reporting of Observational Studies in Epidemiology (STROBE) statement.^23^

### Statistical analysis

Statistical analyses were performed using the R software environment (version R-4.3.1; R Foundation for Statistical Computing, Vienna, Austria). Categorical variables are summarized as counts and percentages. Continuous variables are presented as means with standard deviations (SD) or medians with interquartile ranges (IQR), depending on their distribution. Differences between groups in hospitalization status and other categorical variables were assessed using Fisher’s exact test.

Analyses were performed separately for patients with UC and CD, and then for the combined cohort of all IBD patients. To account for potential confounders, multivariable logistic regression analyses were conducted, including age (<40 vs ⩾40 years), sex, disease duration (<2 vs ⩾2 years), smoking status, IBD type, and biologic use. Results are reported as odds ratios (OR) with 95% confidence intervals (CI). Analyses for each variable included only patients with available data. A two-sided *p*-value <0.05 was considered statistically significant.

## Results

### Study population

Between January 1, 2008 and June 30, 2023, 123 IBD patients in the registry (44 with CD, 79 with UC) were diagnosed with a GI bacterial infection: *Campylobacter* spp. (*n* = 75), *Yersinia* spp. (*n* = 25), *Salmonella* spp. (*n* = 19), or EHEC (*n* = 6). These cases represent 2.4% of all CD and 2.4% of all UC patients in the IBD registry (*N* = 5194; 36% CD, 64% UC). A comparison of baseline characteristics between IBD patients with GI infection and matched controls is shown in Table 1. Among the controls, there were 55 CD patients (45%) and 68 UC patients (55%). In total, data from 246 patients were collected retrospectively and analyzed.

**Table 1.** Comparison of characteristics between IBD patients with GI infection and matched controls.

Characteristic	CD with GI infection	CD control	*p*-Value	UC with GI infection	UC control	*p*-Value
<Patients, *n* (%)	44 (36%)	55 (45%)		79 (64%)	68 (55%)	
Age at time of bacterial infection (years), average ± SD	42.1 ± 12.7	40.1 ± 12.2		40.5 ± 12.7	41.9 ± 12.9	
Female, *n* (%)	19 (43%)	25 (45%)		38 (48%)	31 (46%)	
Male, *n* (%)	25 (57%)	30 (55%)		41 (52%)	37 (54%)	
Montreal classification for CD, *n* (%)						
Inflammatory B1	18 (41%)	20 (36%)				
Stricturing B2	20 (45%)	20 (36%)				
Penetrating B3	6 (14%)	3 (5%)				
Ileum L1 (or + L4)	10 (23%)	12 (21%)				
Colon L2 (or + L4)	9 (20%)	12 (21%)				
Ileocolon L3 (or + L4)	25 (57%)	29 (52%)				
Missing data, location, *n*		16				
Montreal classification for UC, *n* (%)						
Proctitis E1				3 (4%)	10 (15%)	
Left colon E2				17 (21%)	20 (29%)	
Extensive colitis E3				59 (75%)	38 (56%)	
Disease duration (years from diagnosis), median (IQR)	13.5 (2.8–18.5)	10.0 (4.8–16.3)		6.0 (2.0–16.5)	7.0 (3.5–15.0)	
Previous surgical treatment, *n* (%)	18 (41%)	18 (33%)	0.41	7 (9%)	12 (18%)	0.14
Smoking						
Currently smoking, *n* (%)	10 (23%)	11 (20%)	0.80	10 (13%)	10 (15%)	0.81
Never smoked or quit smoking, *n* (%)	32 (73%)	44 (80%)		66 (84%)	58 (85%)	
Missing data, *n*	2			3		
Charlson comorbidity index, *n* (%)						
0	28 (64%)	38 (69%)		48 (61%)	45 (66%)	
1	11 (25%)	13 (24%)		20 (25%)	13 (19%)	
2	3 (7%)	3 (5%)		4 (5%)	7 (10%)	
3	0 (0%)	1 (2%)		6 (8%)	3 (5%)	
4	0 (0%)	0 (0%)		1 (1%)	0 (0%)	
5	2 (5%)	0 (0%)		0 (0%)	0 (0%)	
No pharmacological treatment	5 (11%)	(13%)	>0.99	15 (19%)	14 (21%)	0.84
Aminosalicylates	14 (32%)	19 (35%)	0.83	49 (62%)	44 (65%)	0.86
Thiopurines	18 (41%)	26 (47%)	0.55	21 (27%)	26 (38%)	0.16
Systemic corticosteroids	8 (18%)	8 (15%)	0.78	17 (22%)	5 (7%)	0.020
Calcineurin inhibitors	0 (0%)	1 (2%)	>0.99	2 (3%)	1 (1%)	>0.99
Methotrexate	4 (9%)	9 (16%)	0.38	4 (5%)	2 (3%)	0.69
Biologicals	15 (34%)	17 (31%)	0.83	13 (16%)	8 (12%)	0.48
Anti-TNF	13 (30%)	16 (29%)	>0.99	13 (16%)	7 (10%)	0.34
Vedolizumab	1 (2%)	0 (0%)	>0.99	0 (0%)	1 (1%)	>0.99
Ustekinumab	1 (2%)	1 (2%)	>0.99	0 (0%)	0 (0%)	>0.99
Active IBD (<3 months preceding infection), *n* (%)	15 (34%)	17 (31%)	0.83	30 (38%)	17 (25%)	0.11
Visiting lower hygiene country (<3 months preceding infection), *n* (%)	8 (18%)	NA		15 (19%)	NA	

L4 = upper GI involvement. More than one medication type was used in 17 CD (39%) and 35 UC (44%) patients.

CD, Crohn’s disease; GI, gastrointestinal; IBD, inflammatory bowel disease; IQR, interquartile range; NA, not available; SD, standard deviation; TNF, tumor necrosis factor; UC, ulcerative colitis.

Patients with GI infection were predominantly male (54%) with an average age of 44.1 years. Comorbidities were rare, as CCI was 0 in 62% of the patients. The median disease duration before the positive stool sample was 8.0 years (13.5 years for CD and 6.0 years for UC). There was no pharmacological treatment of IBD for 16% (*n* = 20) of the patients, 51% (*n* = 63) were using aminosalicylates, 32% (*n* = 39) thiopurines, 20% (*n* = 25) systemic corticosteroids, and 23% (*n* = 28) biologicals. None of the patients were treated with JAK inhibitors at the time of the study. The symptoms patients presented during the diagnosis of GI infection are shown in Table 2. All symptoms were new and not attributable to pre-existing IBD activity.

**Table 2.** Gastrointestinal bacterial infection-related symptoms.

Symptoms at diagnosis of infection	CD (*n* = 43)	UC (*n* = 72)	Overall (*n* = 115)
Bloody stools, *n* (%)	7 (6%)	30 (24%)	37 (30%)
Diarrhea, *n* (%)	41 (33%)	69 (56%)	110 (89%)
Abdominal pain, *n* (%)	22 (18%)	43 (35%)	65 (53%)
Nausea, *n* (%)	4 (3%)	5 (4%)	9 (7%)
Vomiting, *n* (%)	3 (2%)	8 (7%)	11 (9%)
Fever, *n* (%)	24 (20%)	36 (29%)	60 (49%)

No symptoms were reported in one CD and seven UC patients. More than one symptom was present in 37 CD (32%) and 62 UC (54%) patients. All symptoms were new and related to the gastrointestinal infection.

CD, Crohn’s disease; UC, ulcerative colitis.

During the 3 months preceding bacterial infection, nearly half of the patients (*n* = 61, 49,6%) had traveled abroad and 24 of them had visited a lower hygiene country (Indian subcontinent, China and South-East Asia, Africa, Southern and Eastern Mediterranean countries including Turkey, the Canary Islands, or Central and South America) compared to the Nordics (Iceland, Finland, Denmark, Norway, Sweden).

Before the GI bacterial infection, 45 patients (37%) had symptoms indicating an active IBD (missing data *n* = 3). Of these patients with previous GI symptoms (indicating active IBD disease), 17 (38%) had medical consultation (emergency department visit or hospitalization), indicating that an active IBD disease before the bacterial infection did not predispose to a more severe infection.

### Comparison between IBD patient group and controls

Up to 37% of patients (*n* = 45) in the GI infection group had an active IBD prior to the infection, and only 28% (*n* = 34) in the controls had IBD symptoms up to 3 months before the evaluation date (*p* = 0.17). In the GI infection group, 45 patients (37%) had an IBD flare 1–6 months after the infection, while only 13 controls (11%) had an IBD flare during the 6-month follow-up (*p* < 0.001). In the GI infection group, 46 patients (37%) had pharmacological treatment intensification 1–6 months after the infection, compared with only 24 cases (20%) in the control group (*p* = 0.003).

Shorter disease duration (<2 years) was significantly associated with higher odds of GI infections (OR 2.34, 95% CI 1.11–5.17, *p* = 0.029). Other variables, including age <40, smoking, female sex, and IBD type, showed no significant associations, Table 3.

**Table 3.** Logistic regression analysis of factors associated with GI bacterial infection.

Variable	OR	95% CI (lower)	95% CI (upper)	*p*-Value
Disease duration <2 years	2.34	1.11	5.17	**0.029**
Age <40 years	0.86	0.51	1.44	0.56
Current smoking	0.95	0.48	1.9	0.89
Female sex	1.04	0.62	1.74	0.89
IBD type (CD = 0; UC = 1)	1.49	0.89	2.52	0.13

Values are presented as OR with 95% CI. Statistically significant *p*-values (<0.05) are shown in bold.

CD, Crohn’s disease; CI, confidence intervals; GI, gastrointestinal; IBD, inflammatory bowel disease; OR, odds ratios; UC, ulcerative colitis.

### Risk factors for emergency department visits and hospitalization

UC patients were more likely to be hospitalized than CD patients (*p* = 0.04). Thirty-four UC patients (28%) and 19 CD patients (15%) visited the emergency department, 26 (21%) and 9 (7%) of whom were hospitalized, respectively.

**Figure 2.**
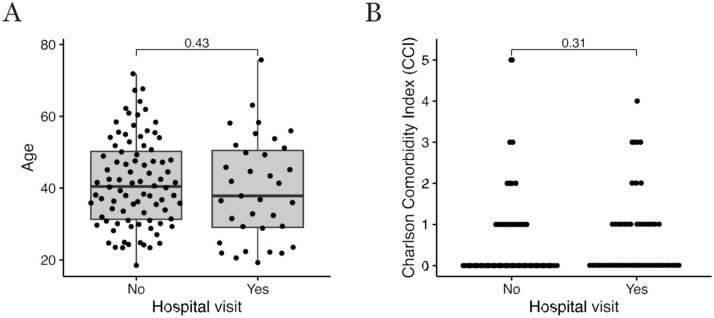
(a) A correlation between age and emergency department visits due to GI infection. (b) A correlation between underlying diseases and emergency department visits due to GI infection. GI, gastrointestinal.

However, there was no difference between colonic/ileocolonic CD patients and distal/extensive UC patients in the need for emergency department visits or hospitalization. In addition, no significant correlation between age and emergency department visits due to GI infection was found (Figure 2(a)). IBD patients were relatively young, and only one patient who was hospitalized was aged over 65 years. Moreover, no significant correlation emerged between disease duration and emergency department visits or hospitalization due to GI infection.

Underlying diseases did not appear to affect the need for emergency department visits (Figure 2(b)). CCI was evaluated and studied in two different groups. When comparing CCI ⩽1 with CCI >1, no significant difference appeared in either emergency department visits or hospitalization between the groups (*p* = 0.60 and *p* = 0.77, respectively).

No significant difference was present in hospital visits between patients who visited a lower hygiene country and those who did not. Furthermore, smoking cessation or nonsmoking status did not protect the patient from a more severe disease; there was no difference in smoking status and the outcome in terms of medical consultation in a hospital setting between CD and UC patients (*p* = 0.28 for CD patients and *p* = 0.33 for UC patients).

Five of the 7 UC patients with a history of surgical treatment were admitted to the hospital, compared with 4 of 18 CD patients. Therefore, previous surgical treatment in UC was considered to be a risk factor for hospitalization (*p* = 0.04), but not in CD (*p* > 1.00).

None of the controls required emergency department visits or hospitalization during the investigated time frame.

### Medication and need for hospitalization

Of the 25 patients using corticosteroids before bacterial infection, 7 (28%) were hospitalized. In addition, one patient visited the emergency department due to severe symptoms but was discharged. Twenty-four patients were on thiopurines, and 16 (67%) of these patients visited the emergency department due to GI infection. Biologicals were used by 28 patients, 12 (43%) of whom visited the emergency department, indicating that thiopurine use may be linked to more severe infections, whereas biologic therapy appears to be associated with fewer emergency department visits.

Corticosteroids, thiopurines, or biologicals were used by 75 patients, of whom 28 (37%) visited the emergency department and 18 (24%) were hospitalized. In patients not using any immunomodulators or suppressants, 25 (52%) visited the emergency department, and 17 (35%) were admitted to a ward. No significant difference between using any of these medications and hospitalization was found. Moreover, out of 103 patients using any IBD medication, 25 were hospitalized (*p* = 0.14), indicating that patients on medical therapy were not more likely to be hospitalized.

The use of antibiotics within 3 months preceding bacterial infection did not have an impact on the need for hospitalization (*p* = 0.62).

**Figure 3.**
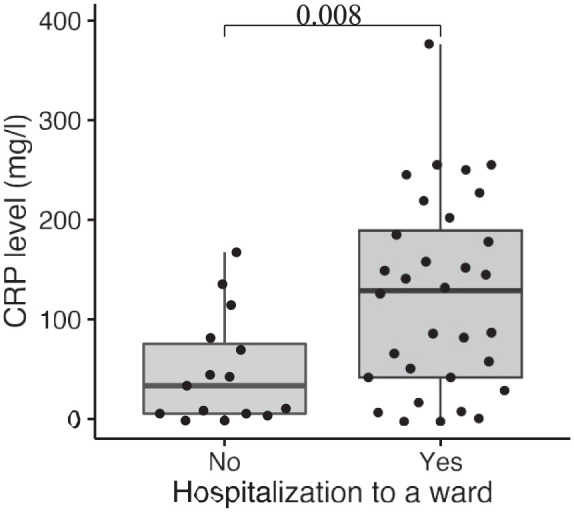
A correlation between CRP and hospitalization due to GI infection. CRP, C-reactive protein; GI, gastrointestinal.

In total, 53 patients (43%) had a medical consultation in the emergency room; 35 patients (66%) were hospitalized, and 18 patients (34%) visited the emergency room but were not admitted to the hospital. During the emergency room visit, C-reactive protein (CRP) was measured in 47 patients. Higher CRP correlated with an increased risk of hospitalization (*p* = 0.008), as shown in Figure 3. Only one patient had a positive blood culture (*Salmonella* spp.).

### Impact of GI infection on medication and disease course

Within 1–6 months after positive bacterial culture, 45 patients (37%) presented symptoms indicating an active IBD, compared with 13 controls (11%), indicating that the bacterial infection predisposed to an IBD flare (*p* < 0.001).

In the GI infection group, escalations in the dosage or a switch to a different medication were made in 46 patients (37%) during 1–6 months after the bacterial GI infection. Biological treatment was initiated in 12 patients, switched to another biological in 4 patients, and the dosage of the biological was increased in 1 patient. After the GI infection, thiopurines were initiated in 11 patients, and the dosage was increased in 2 patients. Methotrexate was initiated in four patients. Among the controls, there were changes made in 24 patients, with a biological initiated in 4 patients. IBD treatment changes were significantly more common in patients with GI infection than in controls (*p* = 0.006). Importantly, there was no significant difference in starting corticosteroids after a bacterial infection in the GI infection group compared with the control group (*p* = 0.36).

The impact of GI bacterial infection on the course of the disease, including hospitalization and changes in medical treatment, is presented in Table 4.

**Table 4.** Outcomes of GI bacterial infections in IBD patients.

Characteristics	CD patients with GI infection (*n* = 44)	CD controls (*n* = 55)	*p*-Value (CD)	UC patients with GI infection (*n* = 79)	UC control (*n* = 68)	*p*-Value (UC)	Overall (*n* = 123)	Overall control (*n* = 123)	*p*-Value
Initiation of antibiotics (up to 1 month from positive stool sample), *n* (%)	36 (82%)	NA		63 (80%)	NA		99 (80%)	NA	
Initiation of corticosteroids (up to 1 month from positive stool sample), *n* (%)	14 (32%)	NA		30 (38%)	NA		44 (36%)	NA	
Hospital visit due to infection, *n* (%)	19 (43%)	0 (0%)		34 (43%)	0 (0%)		53 (43%)	0 (0%)	
Only emergency department visit, *n* (%)	10 (23%)	0 (0%)		8 (10%)	0 (0%)		18 (15%)	0 (0%)	
Admitting to ward, *n* (%)	9 (20%)	0 (0%)		26 (33%)	0 (0%)		35 (28%)	0 (0%)	
Days spent in hospital, median (IQR)	2.0 (1.0–4.0)	0		3.0 (2.0–4.0)	0		3.0 (1.0–4.0)	0	
CRP (at time of positive stool sample), median (IQR)	62.0 (8.3–128.0)	NA		89.0 (22.0–175.0)	NA		83.0 (15.5–157.0)	NA	
IBD flare (1–6 months after positive stool sample), *n* (%)	17 (39%)	6 (11%)	0.002	28 (35%)	7 (10%)	<0.001	45 (37%)	13 (11%)	<0.001
Intensification of any pharmacological treatment (1–6 months after positive stool sample), *n* (%)	16 (36%)	10 (18%)	0.06	30 (38%)	14 (21%)	0.030	46 (37%)	24 (20%)	0.003
Intensification of IBD medication, *n* (%)	11 (25%)	7 (13%)	0.13	26 (33%)	11 (16%)	0.023	37 (30%)	18 (15%)	0.006
Initiation of corticosteroids, *n* (%)	8 (18%)	4 (7%)	0.13	12 (15%)	10 (15%)	>0.99	20 (16%)	14 (11%)	0.36
No pharmacological treatment changes, *n* (%)	28 (64%)	45 (82%)	0.06	47 (59%)	52 (76%)	0.035	75 (61%)	97 (79%)	0.003

Medians for CRP and hospital days are based on patients with available CRP results (*n*=47) and on those who were hospitalized (*n*=53).

CD, Crohns disease; CRP, C-reactive protein; GI, gastrointestinal; IBD, inflammatory bowel disease; IQR, interquartile range; NA, not available; UC, ulcerative colitis.

## Discussion

This study examined GI bacterial infections in a large cohort of IBD patients over an extended period. Our findings indicate that prior IBD medications do not significantly impact the likelihood of hospitalization for IBD patients developing GI bacterial infections. However, patients with high CRP levels or those with UC, as opposed to CD, are more prone to hospitalization. In additionbacterial infections tend to worsen the progression of IBD, often necessitating an escalation in treatment. Notably, our study shows that effective IBD therapies, including immunosuppressive medications, do not increase susceptibility to GI bacterial infections.

There is substantial clinical overlap between GI infections and IBD flares, which may occur independently or simultaneously, complicating diagnostic interpretation and causality assessment. Current clinical guidelines recommend testing for *C. difficile* in all IBD patients presenting with new or worsening diarrhea, and for cytomegalovirus in those with severe, steroid-treated, and refractory disease.^24^ Outside of these recommendations, there is no standardized approach for testing additional GI pathogens during an IBD flare. The adoption of multiplex PCR panels has broadened the detection of various enteric pathogens, although their role in triggering or mimicking IBD flares remains uncertain. In a study by Axelrad et al., nearly 300 multiplex PCR stool tests were performed on IBD patients during flares. After *C. difficile*, the most commonly identified organisms were *Escherichia coli* subtypes (8%) and viral pathogens. Further investigations by the same research group linked enteric infections, altered commensal bacterial function, and IBD pathophysiology.^25,26^

Heightened risk of GI infections may be linked to a combination of different local factors, including gut dysbiosis, impaired repair of the gut epithelial barrier, dysregulated immune responses, and chronic intestinal inflammation. These elements are also central to the pathogenesis of IBD, especially in individuals with genetic susceptibility.^24^ Moreover, several studies have investigated whether GI infections in IBD patients increase the risk of hospitalization, with various factors such as age, sex, comorbidities, corticosteroid use, and IBD type influencing this risk. These studies have similarly shown that GI infections are more common in people with IBD.^27–30^

Hospitalization risk increases significantly in those with severe IBD, especially when treated with corticosteroids.^31^ Furthermore, elderly patients, individuals with multiple comorbidities,^32^ and, in contrast to our study, those with CD versus UC tend to experience more frequent and severe infections, leading to a higher likelihood of hospitalization.^33^ It is suggested that a complex interaction between GI infection, IBD characteristics, and individual patient factors influences the likelihood of hospitalization.^30^ It has also been suggested that UC patients, due to their disrupted immune–microbiota axis and less resilient microbiota, may represent a vulnerable population for GI bacterial infections such as *E. coli*.^34^ While our findings align with the recognized impact of disease severity and systemic inflammation (e.g., elevated CRP) on hospitalization risk, they differ in suggesting that prior IBD medication use, including immunosuppressants, does not significantly contribute to this risk.

Finland has a relatively high prevalence of IBD (1%)^35^ and is known for its standardized treatment protocols, equitable access to care, effective medication, active involvement of IBD-specialized nurses, and strong patient support organizations. These factors likely contribute to the low detection rates of GI infections in IBD patients over an extended period, as patients are well-informed about the potential risks of opportunistic infections associated with their immunosuppressive medications.

In contrast to our findings, Kirchgesner et al.^36^ reported that patients receiving combination therapy, antitumor necrosis factor (TNF) monotherapy, or thiopurine monotherapy had a higher risk of serious infections than those not treated with thiopurines or anti-TNFs. Many earlier observational studies examining infection risk associated with these treatments in IBD did not simultaneously account for disease activity and corticosteroid use over time. Yet, both disease activity and corticosteroid exposure are significant predictors of infection, as demonstrated by a U.S. study using data from the TREAT (Crohn’s Therapy, Resource, Evaluation and Assessment Tool) registry,^37^ and these factors can influence treatment adjustments and infection risk.

Our study has some limitations. First, diagnostic methods varied over time—both stool culture and nucleic acid amplification tests were used—so a single uniform approach was not applied across all patients. Second, due to the retrospective design, incomplete data in patient charts made it challenging to identify variables linked to unfavorable outcomes and an increased risk of hospitalization. In addition, in some cases, it was unclear whether patients adhered to their prescribed medications. Furthermore, only clinical disease activity scores were available; endoscopic or biomarker-based data were inconsistently documented and therefore not systematically included. Finally, there were relatively few IBD patients with GI bacterial infections over the 15-year study period. Despite these limitations, we believe this study, with its extensive 15-year data collection period, provides valuable insights into real-world clinical practices and offers important data on GI bacterial infections within the IBD population.

Based on our results, previous surgical treatment, specifically proctocolectomy, in UC was associated with a negative outcome, such as more severe disease and need for hospitalization. Moreover, IBD patients who had GI bacterial infection were more likely to have a flare in 1–6 months after the infection was diagnosed, resulting in intensification of their IBD medication. This highlights the importance of taking this into account when following up patients. Surprisingly, the use of corticosteroids was not increased after bacterial infection compared with controls.

## Conclusion

Based on our study, effective IBD medication does not significantly increase the risk of severe GI infections. However, it is important to rule out GI bacterial infections in case of an IBD flare, as they may influence the subsequent course of the disease, requiring an escalation in medication. Patients may benefit from careful follow-up for 6 months after GI infection.

## Supplemental Material

sj-docx-1-tag-10.1177_17562848251386318 – Supplemental material for Gastrointestinal bacterial infections precede disease activation and treatment intensification in patients with inflammatory bowel disease Supplemental material, sj-docx-1-tag-10.1177_17562848251386318 for Gastrointestinal bacterial infections precede disease activation and treatment intensification in patients with inflammatory bowel disease by Merit Kase, Krista Johanna Vitikainen, Clas-Göran af Björkesten, Veli-Jukka Anttila, Leo Meriranta, Perttu Arkkila and Pauliina Molander in Therapeutic Advances in Gastroenterology

## Declarations

**Ethics approval and consent to participate:** Research study permission (HUS/155/2021) was received from the institutional review board at Helsinki University Hospital. Being a retrospective, non-interventional patient chart review study, no ethics committee application was required. Informed consent was not obtained, as patients were not contacted, and all data were analyzed in accordance with applicable data protection regulations.

**Consent for publication:** Patients were not involved in the design, execution, or dissemination plans of this research. Patient consent was not required.

**Availability of data and materials:** The data may be requested from the corresponding author.
